# Localization of protein kinase C ε to macrophage vacuoles perforated by *Listeria monocytogenes* cytolysin

**DOI:** 10.1111/j.1462-5822.2007.00903.x

**Published:** 2007-07-01

**Authors:** Lee M Shaughnessy, Peter Lipp, Kyung-Dall Lee, Joel A Swanson

**Affiliations:** 1 Department of Microbiology and Immunology, University of Michigan Medical School Ann Arbor, MI 48109, USA.; 2 Institute for Molecular Cell Biology, Saarland University Homburg, Germany.; 3 Department of Pharmaceutical Sciences, College of Pharmacy, University of Michigan Ann Arbor, MI 48109, USA.

## Abstract

Three proteins secreted by *Listeria monocytogenes* facilitate escape from macrophage vacuoles: the cholesterol-dependent cytolysin listeriolysin O (LLO), a phosphoinositide-specific phospholipase C (PI-PLC) and a broad-range phospholipase C (PC-PLC). LLO and PI-PLC can activate several members of the protein kinase C (PKC) family during infection. PKCε is a novel PKC that contributes to macrophage activation, defence against bacterial infection, and phagocytosis; however, a role for PKCε in *Lm* infections has not been described. To study PKCε dynamics, PKCε-YFP chimeras were visualized in macrophages during *Lm* infection. PKCε-YFP was recruited to forming vacuoles during macrophage phagocytosis of *Lm* and again later to fully formed *Lm* vacuoles. The PKCε-YFP localization to the fully formed *Lm* vacuole was LLO-dependent but independent of PI-PLC or PC-PLC. PKCε-YFP recruitment often followed LLO perforation of the membrane, as indicated by localization of PKCε-YFP to *Lm* vacuoles after they released small fluorescent dyes into the cytoplasm. PKCε-YFP recruitment to vesicles also followed phagocytosis of LLO-containing liposomes or osmotic lysis of endocytic vesicles, indicating that vacuole perforation by LLO was the chief cause of the PKCε response. These studies implicate PKCε in a cellular mechanism for recognizing damaged membranous organelles, including the disrupted vacuoles created when *Lm* escapes into cytoplasm.

## Introduction

Macrophages are essential for clearing *Listeria monocytogenes* (*Lm*) infections in mice (Mackaness, 1962; Adams and Hamilton, 1984; Kiderlen *et al*., 1984; Pamer, 2004). *Lm* enters macrophages by phagocytosis and then escapes from phagosomal vacuoles into the cytosol, where it can replicate and invade neighbouring cells. *Listeria* secretes a cholesterol-dependent cytolysin (CDC), listeriolysin O (LLO), which is necessary for escape from the phagosome into the cytosol (Portnoy *et al*., 1988; Cossart *et al*., 1989; Gedde *et al*., 2000). *Lm* also secretes two phospholipases C which have minor roles in escape (Smith *et al*., 1995): a phosphatidylinositol-specific phospholipase C (PI-PLC) and a broad-range phospholipase C (PC-PLC).

In addition to their involvement in bacterial escape, LLO and the bacterial phospholipases C (PLCs) induce signalling from the phagosome (Goldfine and Wadsworth, 2002). CDCs, including LLO, induce secretion of TNFα and IL-6 and activate macrophages by inducing iNOS expression (Park *et al*., 2004). In addition, Goldfine and colleagues identified a LLO-mediated activation of host PLC and phospholipase D following *Lm* infection of macrophages (Goldfine *et al*., 2000).

Protein kinases C (PKCs) are phospholipid-dependent, serine/threonine protein kinases whose 11 isozymes are placed into subfamilies based upon their cofactor requirements for activation (Newton, 2001). Conventional PKCs [α, β(I and II), γ] are activated by calcium (Ca^2+^), diacylglycerol (DAG), and phosphatidylserine (PS) or anionic phospholipids. Novel PKCs (δ, ε, η, θ) are activated by DAG and PS. Atypical PKCs (ζ, ι/λ) are activated by PS. LLO and PI-PLC activate recruitment of PKCδ to plasma membranes and PKC βII to early endosomes (Wadsworth and Goldfine, 2002). Inhibition of PKC βII increased *Lm* phagocytosis and decreased escape from vacuoles (Wadsworth and Goldfine, 2002), indicating that *Lm* exploits host PKC βII activity during infection.

Protein kinases C regulate a variety of cellular processes including cytoskeleton rearrangements and immune cell signalling (Tan and Parker, 2003). Signalling through the Fc-receptor induces PLC-mediated hydrolysis of phosphatidylinositol 4,5 bisphosphate, generating inositol-1,4,5 trisphosphate, which increases [Ca^+2^]_i_, and DAG, second messengers that activate conventional PKCs. PKCε is recruited to IgG-opsonized particles in forming phagosomes and is necessary for FcγR-mediated phagocytosis in macrophages (Larsen *et al*., 2000; 2002). PKCε has been implicated in innate immunity through its role in macrophage activation (Castrillo *et al*., 2001). PKCε also upregulates the expression of iNOS and subsequent NO production (Diaz-Guerra *et al*., 1996). Many bacteria (*Listeria*, *Bacillus* and *Staphylococcus* species) also secrete phospholipases, including PI-PLC and PC-PLC, which can produce DAG and potentially recruit host PLC for subsequent signal transduction.

Upon phosphorylation at three residues in the catalytic kinase domain, PKCs become mature; a prerequisite for binding to second messengers and activation (Keranen *et al*., 1995; Cenni *et al*., 2002). Mature but unactivated PKCs are localized in the cytosol; upon activation they translocate to the membrane (Newton, 2001). Novel PKCs translocate to the plasma membrane in response to DAG formation (Stahelin *et al*., 2005). Once bound to DAG, novel PKCs bind to anionic phospholipids which allows the pseudosubstrate domain to be released and PKC to phosphorylate its substrate. Inside cells, compartmentalization in space or time targets the different PKC isoforms to different signalling pathways. Targeting and substrate specificity for each PKC isoform depends upon subcellular and tissue localization (Akita, 2002).

Castrillo *et al*. demonstrated that PKCε is necessary for lipopolysaccharide-induced macrophage activation and defence against infection by *Escherichia coli* and *Staphylococcus aureus* (Castrillo *et al*., 2001). Macrophages from PKCε –/– mice showed reduced ability to produce nitric oxide, TNF-α, and IL-1β in response to lipopolysaccharide and IFN-γ (Castrillo *et al*., 2001). The Toll-like receptor 4 (TLR4) adapter molecule TRAM was recently identified as a specific substrate for PKCε (McGettrick *et al*., 2006). Phosphorylation of TRAM by PKCε is necessary for TRAM-mediated TLR signalling (McGettrick *et al*., 2006). These observations indicate roles for PKCε in TLR signalling and the early signalling events necessary for macrophage activation (Aksoy *et al*., 2004; McGettrick *et al*., 2006). There is no evidence thus far that PKCε is recruited to membranes during bacterial entry.

To examine the role of PKCε in macrophage responses to infection, we expressed in macrophages a fluorescent chimera of PKCε, PKCε-YFP, and analysed its intracellular dynamics in macrophages during *Lm* infection. We identified a LLO- and PLC-independent accumulation of PKCε upon *Lm* entry into macrophages, as well as a later, LLO-dependent concentration of PKCε on vacuoles following perforation of the *Lm* vacuole membrane. This later PKCε recruitment could also be elicited by liposomes containing purified LLO or by osmotic lysis of endosomes, indicating a role for PKCε in the detection of damaged membrane organelles in macrophages.

## Results

### Protein kinase C ε is recruited to *Lm*-associated membranes in macrophages

**Fig. 1:**
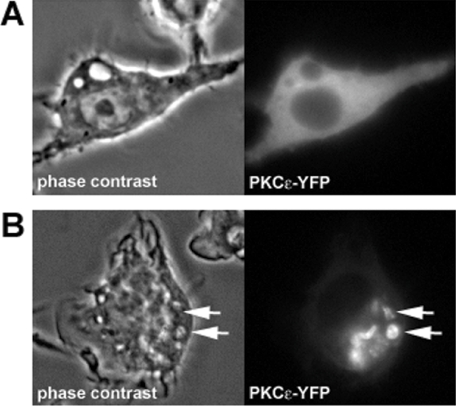
PKCε is recruited to *Lm*-associated membranes in macrophages. Macrophages expressing PKCε-YFP were either (A) uninfected or (B) infected with wild-type *Lm*. Representative phase-contrast and YFP images are shown. Arrows point to wild-type *Lm* and the recruitment of PKCε-YFP to associated membranes.

**Fig. 2:**
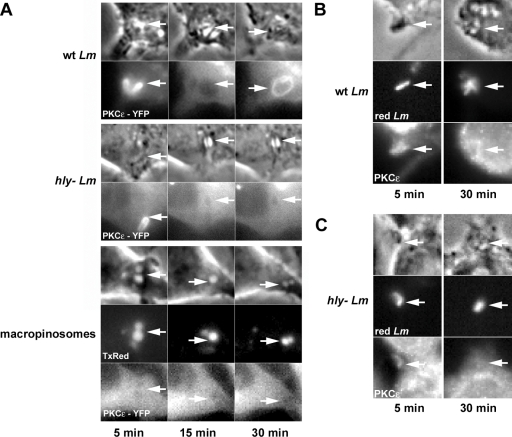
Wild-type *Lm* recruits PKCε to the phagosome. A. Macrophages expressing PKCε-YFP were either infected with wild-type *Lm* or *hly Lm*, or were labelled by endocytosis of Texas Red dextran (TxRed). Phase-contrast (upper panel), YFP and TRDx images were collected at 5 min (left image), 15 min (centre image) and 30 min (right image) after addition of *Lm* or dye. Arrows point to the vacuole. B and C. Distributions of endogenous PKCε. RAW macrophages were infected with either wild-type (B) or *hly* (C) *Lm* (SNARF-labelled; red) and PKCε was visualized by immunofluorescence and stained with Alexa Fluor 488-labelled secondary antibody.

In resting cells, PKCε-YFP was mostly distributed uniformly throughout the macrophage cytoplasm, with minor perinuclear localization (Fig. 1A). After wild-type *Lm* infection of macrophages, PKCε-YFP robustly localized to membranes associated with the bacteria (Fig. 1B). We asked if *Lm* recruitment of PKCε to vacuolar membranes is affected by LLO. RAW 264.7 macrophages expressing PKCε-YFP were infected with wild-type *Lm* or *hly* (LLO-deficient) *Lm,* and localization of PKCε-YFP was analysed by time-lapse microscopy of live cells. Shortly after *Lm* entry, PKCε was recruited to both wild-type and *hly Lm*-associated membranes (Fig. 2A; 5 min). Time-lapse movies showed that the membranes that recruited PKCε-YFP during entry were close to the bacteria, consistent with a role for PKCε in *Lm* phagocytosis (Fig. 2A; Time 5). PKCε accumulation at *Lm*-containing phagosomes was biphasic, occurring during phagosome formation (5 min) and again around the fully formed *Lm* vacuoles. PKCε translocation was specific for *Lm* vacuoles; it did not translocate to macropinosomes loaded with only Texas Red dextran (TRDx; 10 000 MW dextran) (Fig. 2A). This second recruitment of PKCε-YFP was dependent upon the presence of LLO, as PKCε-YFP localized to wild-type *Lm* vacuoles but not *hly Lm* vacuoles (Fig. 2A, Time 30). Labelling of endogenous PKCε by immunofluorescence showed similar localization patterns: wild-type (Fig. 2B) and *hly* (Fig. 2C) *Lm* recruited PKCε upon entry (5 min), but at 30 min only vacuoles with wild-type *Lm* contained PKCε.

### *Lm* PLCs do not affect PKCε recruitment to vacuolar membranes

**Fig. 3:**
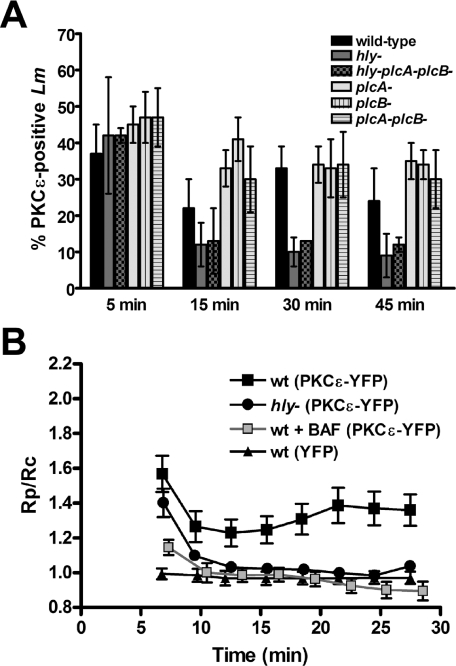
Conditions affecting PKCε recruitment to *Lm* vacuoles. A. Quantitative analysis of the timing of PKCε localization to *Lm*. Macrophages were infected for 3 min with the indicated strains of bacteria, then were washed and fixed at 5, 15, 30 and 45 min after infection. Bacteria were labelled with DAPI and scored for colocalization with PKCε-YFP. Fifty bacteria were counted in triplicate in three different experiments. B. Macrophages were transfected with plasmids for PKCε-YFP and CFP (or YFP and CFP for the negative control; *n* = 10). Transfected macrophages, untreated or treated with 500 nM bafilomycin A_1_ (BAF; *n* = 10), were infected with either wild-type (*n* = 18) or *hly Lm* (*n* = 11) for 3 min. Time-lapse phase-contrast, CFP and YFP images were taken of *Lm* phagosomes every 30 s for 30 min, and ratio images were prepared (YFP/CFP). The ratio of YFP/CFP in the phagosome (Rp) was divided by the ratio of YFP/CFP in the entire cell (Rc) to measure recruitment of PKCε-YFP to the phagosome (Rp/Rc).

As PKCε associates with membranes via binding to DAG as well as other lipids, we tested the hypothesis that PI-PLC or PC-PLC from *Lm* generates lipids for PKCε docking to *Lm* vacuoles. Specifically, we quantified the percentage of *Lm* vacuoles that recruited PKCε-YFP after infection with wild-type *Lm* or *Lm* mutants deficient in LLO (*hly-*), PI-PLC (*plcA-*), PC-PLC (*plcB-*) or combinations thereof (*hly plcA-plcB-*, *plcA-plcB-*). At 5 min after infection, similar percentages were observed for wild-type (37%), *hly* (42%), *plcA-* (45%), *plcB-* (47%), *plcA-plcB-* (47%), and *hly plcA-plcB-* (42%) *Lm* phagosomes, indicating that LLO and PLCs were not necessary for PKCε recruitment during bacterial entry (Fig. 3A).

At later time points, PKCε translocated more frequently to phagosomes containing LLO-expressing *Lm* (Fig. 3A; Time 15, 30 and 45 min). Approximately 25–35% of LLO-expressing *Lm* (wild-type, *plcA-*, *plcB-*, *plcA-plcB-*) recruited PKCε-YFP 15–45 min after infection, whereas only 10% of LLO– *Lm* (*hly* and *hly plcA-plcB-*) recruited PKCε-YFP at these later times. This indicated that the bacterial PLCs were not producing the DAG that recruited PKCε to the vacuolar membrane. Interestingly, at 15 min, the measurements for *plcB-*, *plcA-*, *plcA-plcB-*, and *hly Lm* were significantly different from that of wild-type *Lm* (*P* < 0.05). This suggests the PLCs may modulate the PKCε response.

### Protein kinase C ε recruitment to the *Lm* vacuole membrane is dependent upon LLO and vacuolar acidification

We quantified the levels of PKCε translocated to LLO+ and LLO– *Lm* vacuoles (Fig. 3B). LLO– *Lm* vacuoles were not completely devoid of PKCε recruitment, indicating that PKCε was recruited to *Lm* vacuoles at low levels but that LLO somehow enhanced that recruitment. Macrophages coexpressing PKCε-YFP and CFP were infected with wild-type or *hly Lm*. Phase-contrast, YFP and CFP images were taken of phagosomes at regular intervals. Ratio images (YFP/CFP) were calculated, and the ratio of YFP/CFP of the phagosome (Rp) was divided by the ratio of YFP/CFP in the whole cell (Rc). Rp/Rc values greater than 1.0 indicated YFP chimera recruitment to vacuoles (Fig. 3B; triangles) (Henry *et al*., 2004). Both wild-type and *hly Lm* showed similar high amounts of PKCε-YFP recruited upon entry (Time 5), with Rp/Rc values of 1.6 and 1.4 respectively. The second recruitment to the wild-type *Lm* phagosome (Rp/Rc values of ∼1.3–1.4) was maximal at 22 min and was significantly higher than seen on *hly Lm* phagosomes (Rp/Rc values ∼1.05) (*P* < 0.01).

Addition of bafilomycin A_1_ to macrophages increases the pH of endocytic compartments by inhibition of the proton ATPase, and consequently inhibits LLO pore-forming activity (Beauregard *et al*., 1997; Shaughnessy *et al*., 2006). We asked if inhibiting LLO activity with bafilomycin A_1_ would affect PKCε recruitment to the *Lm* vacuole. Treatment of macrophages with bafilomycin A_1_ before and during infection reduced wild-type *Lm* activation of PKCε to baseline levels (Fig. 3B). This indicated that LLO pore-forming activity was necessary for the late PKCε recruitment to the *Lm* vacuole.

### Protein kinase C ε is recruited to perforated *Lm* vacuoles

We previously showed by measuring the sequential release of small [Lucifer Yellow (LY); 522 MW] and large (TRDx; 10 000 average MW) fluorescent probes from *Lm* vacuoles that LLO-expressing (LLO+) *Lm* perforate vacuolar membranes (Shaughnessy *et al*., 2006). In the previous study, 50% of LLO+ *Lm* perforated the vacuole, which was characterized by the selective release of LY from the vacuole or the sequential release of LY then TRDx (Shaughnessy *et al*., 2006). The other 50% of LLO+ *Lm* vacuoles and 100% of LLO– *Lm* vacuoles did not perforate the vacuole (Shaughnessy *et al*., 2006). We define perforation of the *Lm* vacuole as the differential release of LY and TRDx; either by loss of LY only or by sequential loss of LY and then TRDx.

**Fig. 4:**
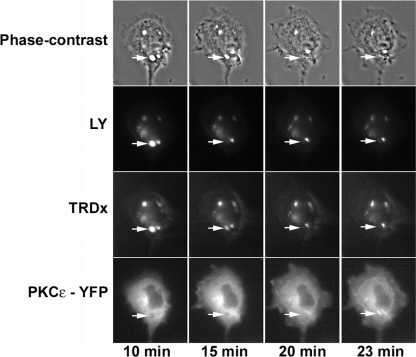
PKCε localizes after perforation of the *Lm* phagosome. PKCε-YFP-expressing macrophages were infected with wild-type *Lm* in the presence of LY and TRDx. Time-lapse phase-contrast and fluorescence images of YFP, LY and TRDx in *Lm* phagosomes were taken at regular intervals. Shown are representative images taken at 10, 15, 20 and 23 min after infection. Arrow points to a *Lm* phagosome that loses LY and TRDx then acquires PKCε-YFP.

We adapted this method to ask if vacuoles recruiting PKCε were first perforated by LLO. Macrophages expressing PKCε-YFP were infected with wild-type *Lm* in the presence of LY and TRDx. The fluorescence of LY, TRDx, and PKCε-YFP was then imaged in the *Lm* vacuoles over time. Time-lapse fluorescence revealed that PKCε-YFP translocation to wild-type *Lm* vacuoles occurred after perforation of the vacuole. That is, the sequential loss of fluorescence of LY and TRDx from the phagosome was apparent 10–15 min after *Lm* addition whereas translocation of PKCε was not detected until approximately 23 min (Fig. 4). As in previous studies (Shaughnessy *et al*., 2006), half of wild-type *Lm* vacuoles (6 of 11 recorded events) showed perforation. Of the six phagosomes that perforated, five later recruited PKCε-YFP (Fig. 4). Of the five events in which *Lm* did not lose LY and TRDx, only one showed PKCε-YFP translocation to the vacuole. No vacuoles perforated after recruitment of PKCε-YFP. A Pearson's chi-squared test applied to these results indicated that the correlation between perforation and PKCε recruitment was significant (*P* = 0.0356). These results indicated that LLO pore-forming activity is necessary for accumulation of PKCε.

### Protein kinase C ε is recruited by vacuolar LLO

**Fig. 5:**
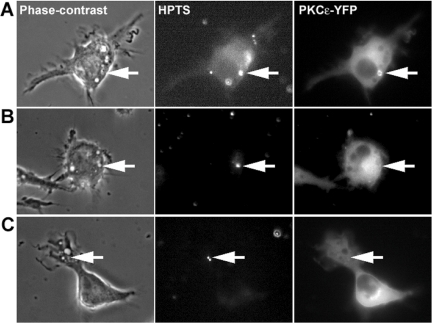
PKCε localizes to perforated compartments. PKCε-YFP-expressing macrophages were infected with liposomes containing (A) HPTS and LLO, (B) HPTS and hiLLO, or (C) HPTS. Shown are representative phase-contrast and fluorescence HPTS and YFP images with arrows pointing to endosomes containing liposomes. PKCε-YFP was only recruited to vacuoles containing functional LLO.

We next asked if LLO activity in the vacuole was sufficient to recruit PKCε. Macrophages were allowed to phagocytose pH-sensitive liposomes containing LLO. These liposomes (phosphatidylethanolamine–cholesterylhemisuccinate) become unstable at pH < 6.0 and release their contents, allowing delivery of encapsulated molecules, including LLO, into the endocytic compartment (Lee *et al*., 1996). Macrophages expressing PKCε-YFP were fed pH-sensitive liposomes in which LLO was co-encapsulated with a small fluorescent dye, 8-hydroxypyrene-1,3,6-trisulfonic acid (HPTS). Delivery of LLO (Fig. 5A), but not heat-inactivated LLO (hiLLO) (Fig. 5B) or HPTS alone (Fig. 5C), into the endocytic compartment caused PKCε-YFP translocation to the phagosomes. LLO and HPTS liposomes recruited PKCε-YFP to 5% of the vacuoles, whereas liposomes containing HPTS alone or hiLLO plus HPTS never recruited PKCε-YFP (50 vacuoles were counted for three separate experiments). This indicated that endosomal LLO was sufficient to recruit PKCε to compartments and suggests that PKCε recruitment signals vacuole perforation.

### Protein kinase C ε is recruited to osmotically lysed endosomes

**Fig. 6:**
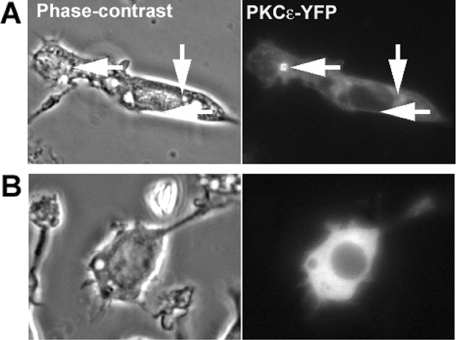
PKCε localizes to osmotically lysed endosomes. Representative phase-contrast and YFP images of PKCε-YFP-expressing macrophages exposed to either (A) hypertonic media containing polyethylene glycol 1000, which lyses endosomes osmotically, or (B) hypertonic media with no polyethylene glycol 1000, which does not lyse endosomes.

The results to this point are consistent with two models: (i) LLO is required for PKCε recruitment or (ii) LLO is not required and other signals that induce membrane damage will serve equally for the same task. To distinguish between these possibilities, we determined the effect of osmotic lysis of endosomes on PKCε translocation. Endosomes in RAW macrophages expressing PKCε-YFP were ruptured by osmotic lysis, a commonly used method to deliver antigens from endosomes into the cytosol (Okada and Rechsteiner, 1982; Moore *et al*., 1988). Briefly, macrophages were allowed to endocytose hypertonic medium, and then hypotonic medium was added, causing endosome rupture and release of their contents into cytoplasm. Finally an isotonic solution was added to allow macrophage recovery. When this procedure was performed with macrophages expressing PKCε-YFP, multiple vacuoles recruited PKCε-YFP to their membranes (Fig. 6A). Omission of 10% polyethylene glycol 1000 from the hypertonic medium does not allow cytoplasmic release of macromolecules (Okada and Rechsteiner, 1982; Moore *et al*., 1988). In macrophages exposed to this control condition, PKCε-YFP no longer translocated to endocytic membranes (Fig. 6B). Moreover, isotonic medium and hypotonic medium alone also did not cause PKCε-YFP to translocate to endocytic membranes (data not shown). This indicates that PKCε recruitment to *Lm* vacuoles is part of a cytoplasmic signalling mechanism that recognizes damaged organelles.

## Discussion

This work demonstrates that PKCε localizes twice to *Lm*-associated membranes during *Lm* infection of macrophages. PKCε localization during *Lm* entry is independent of LLO and the bacterial PLCs. Later, PKCε localizes to the *Lm*-containing vacuole after perforation by LLO. This indicates a role for PKCε in recognizing damaged *Lm* vacuoles.

### A role for PKCε in *Lm* pathogenesis

Protein kinase C ε localized to *Lm* upon entry into macrophages, independently of LLO or the bacterial PLCs. This localization is likely related to that described for FcγR-mediated phagocytosis of IgG-opsonized particles (Diaz-Guerra *et al*., 1996; Larsen *et al*., 2000; 2002). Inhibition of PKCε slowed the rate of FcγR-mediated phagocytosis and decreased NO production (Larsen *et al*., 2000; 2002). NO production is important for the clearance of *Lm* in activated macrophages. It is possible that PKCε recruitment during *Lm* entry signals the induction of NO production near the vacuole.

The activity of LLO inside *Lm* vacuoles stimulated a second localization of PKCε. The recruitment of PKCε to wild-type *Lm* vacuoles followed LLO-mediated vacuole perforation and required LLO activity. This LLO-dependent recruitment of PKCε-YFP suggests either that *Lm* exploits host cell signalling mechanisms or that the host uses PKCε as a means to signal for the presence of a damaged vacuole. The low level of PKCε recruitment to vacuoles without LLO may indicate the normal level of PKCε that is recruited to phagosomes which is necessary for defence against bacterial infection.

Our hypothesis at the outset of these studies was that *Lm* PLCs recruit PKCε to the vacuolar membrane. Both host cells and *Lm* produce PLCs that hydrolyse phospholipids, releasing DAG on membranes. Therefore, both the host and *Lm* are equipped to activate PKCε. It was previously reported that *Lm* PLCs activate host PKCs (Goldfine and Wadsworth, 2002; Wadsworth and Goldfine, 2002). However, as we did not see a decrease in PKCε recruitment relative to wild-type *Lm* after infection with *Lm* mutants *plcA-*, *plcB-* or the double mutant *plcA-plcB-*, we conclude that they have no role in activating PKCε. These results therefore implicate host PLCs for PKCε activation (Goldfine *et al*., 2000). DAG, localized in macrophages expressing the DAG-binding domain from PKCδ (C1δ-GFP), appeared on *Lm*-associated membranes both during entry and later on the vacuole membrane (data not shown).

Cholesterol-dependent cytolysins could form pores that specifically translocate proteins into the host cell. This is supported by studies of another CDC, streptolysin O, which secretes an effector molecule through its pore into the host cell (Madden *et al*., 2001). Likewise, it was hypothesized that LLO is a specific translocator for the *Lm*-secreted proteins, PI-PLC and PC-PLC (Goldfine and Wadsworth, 2002). Our studies indicate no role for the bacterial PLCs in the activation of PKCε, despite the fact that PKCε recruitment occurs after membranes have been permeabilized by LLO.

### Listeriolysin O: an activator of signalling events from the phagosome

The substrate of PKCε on the *Lm* vacuole is not yet known, so it is unclear what downstream molecules are involved. LLO and other CDCs have previously been shown to mediate host signalling pathways. CDCs activate TLR4 signalling events, including induction of TNFα, IL-6 and iNOS (Park *et al*., 2004). PKCε has also been implicated in TLR signalling (Aksoy *et al*., 2004; McGettrick *et al*., 2006). Recently, the TLR4 adaptor molecule TRAM was identified as a specific substrate for PKCε; phosphorylation of TRAM by PKCε was necessary for downstream TRAM-mediated TLR4 signalling (McGettrick *et al*., 2006). It is unknown whether TLR4 is involved in recognition of *Lm*; however, activation of MyD88 (another TLR4 adaptor molecule) is essential for the innate immune defence against *Lm* (Edelson and Unanue, 2002; Seki *et al*., 2002; Pamer, 2004). Therefore, it is possible that *Lm* is recognized by TLR4 and signals through TRAM and PKCε on the *Lm* vacuoles. Future studies should determine if PKCε recruitment to the *Lm* vacuole enhances signalling by TLRs or other pattern-recognition receptors.

### The role of PKCε in cellular responses to membrane damage

It is unknown what happens to damaged compartments and how the host cell either repairs or recycles the membrane. Damaged organelles could be sealed and ready to use again or, if the membrane is beyond repair, the membrane may be sequestered and degraded by autophagy. PKCε signalling may be involved in the breakdown and recycling of damaged membrane.

The PKCε recruitment to *Lm* vacuoles may indicate a general mechanism for recognizing damaged organelles. What could be the consequences of recruiting PKCε to a damaged organelle? Many viruses and intracellular pathogens reach cytoplasm by disrupting vesicles that contain them after endocytosis by host cells. For *Lm*, inflammatory responses of infected macrophages and dendritic cells require LLO (Vazquez *et al*., 1995; Brzoza *et al*., 2004). Perhaps inflammatory or immune responses of host cells to invasive microbes are activated by PKCε binding to lipids or proteins which normally reside in the outer (luminal) leaflet of endocytic vesicle membranes, but which are exposed to cytoplasm when the integrity of the bilayer is compromised.

## Experimental procedures

### Reagents

The fluorophores Texas Red phalloidin, TRDx (MW = 10 kDa), LY, HPTS, and 4′,6-diamindino-2-phenylindole (DAPI) were obtained from Molecular Probes (Eugene, OR). Bafilomycin A_1_ was obtained from Calbiochem (La Jolla, CA).

### Bacterial strains

The *Lm* wild-type strain DP-L10403, *hly* deletion strain DP-L2161, *plcA-* deletion strain DP-L1552, *plcB-* deletion strain DP-L1935, *plcA-plcB-* deletion strain DP-L1936, *hly plcA-plcB-* deletion strain DP-L2319 used in this study were gifts from Daniel Portnoy (University of California, Berkeley).

### Bacterial preparation

*Listeria monocytogenes* were grown overnight at room temperature in Brain Heart Infusion broth. They were subcultured the next day and grown for ∼1 h to an OD_600_ of 0.500 at 37°C. Subcultured bacteria (1 ml) were washed three times in 1 ml Ringer's buffer (RB; 155 mM NaCl, 5 mM KCl, 2 mM CaCl_2_, 1 mM MgCl_2_, 2 mM NaH_2_PO_4_, 10 mM Hepes and 10 mM glucose, pH 7.2) followed by centrifugation (4500 *g*). Where indicated, bacteria were pre-labelled with SNARF-1, carboxylic acid, acetate succinimidyl ester (Molecular Probes). Washed bacteria were labelled with 3 μl ml^−1^ SNARF-1 solution in DMSO for 15 min at 37°C with shaking, then washed four times with 1 ml RB before use.

### Macrophage preparation

RAW 264.7 macrophages were obtained from ATCC (Manassas, VA) and grown in Advanced Dulbecco's modified Eagle's medium (DMEM, Invitrogen, Carlsbad, CA), with 2% heat-inactivated FBS (Invitrogen), 100 unit ml^−1^ of penicillin/streptomycin mixture (Sigma Chemical, St Louis, MO), and l-glutamine, at 37°C with 5% CO_2_. Cells were plated the day before the experiment onto 25 mm coverslips, in 6 well plates, at 3 × 10^5^ cells well^−1^. Macrophages were transfected with plasmids for fluorescent chimera expression using FuGENE 6 transfection reagent, according to the manufacturer's protocol (Roche Diagnostics, GmbH, Mannheim, Germany).

### Fixed cell assay and immunofluorescence

Macrophages were infected with *Lm* (multiplicity of infection ∼1) for 3 min. Coverslips were washed with RB and incubated with DMEM, 10% FBS, and 25 μg ml^−1^ gentamicin. Coverslips were fixed with cytoskeletal fix (30 mM Hepes, 10 mM EGTA, 0.5 mM EDTA, 5 mM MgSO_4_, 33 mM potassium acetate, 5% polyethylene glycol 400, and 4% paraformaldehyde) at either 5, 15, 30 or 45 min after infection. Cells were rinsed with phosphate-buffered saline (PBS) and 2% goat serum, permeabilized with 0.3% Triton X-100 in PBS, and incubated for 15 min in PBS and 2% goat serum plus DAPI (2 μg ml^−1^ from a 100 μg ml^−1^ stock in water). For immunofluorescence staining, non-transfected macrophages were incubated with 1:50 dilution of mouse monoclonal anti-PKCε antibody (Santa Cruz Biotechnology, Santa Cruz, CA) in 2% goat-serum overnight at 4°C and rinsed three times for 5 min with 2% goat-serum. A 1:1000 dilution of Alexa Fluor 488-labelled secondary antibody (Molecular Probes) in 2% goat-serum was incubated for 1 h at 37. Coverslips were mounted on glass slides containing Prolong Antifade Gold (Molecular Probes). For each coverslip, 50 *Lm* were scored for the presence of PKCε localization.

### Ratiometric imaging

Macrophages expressing PKCε-YFP (or untagged YFP when indicated) and CFP were infected with *Listeria* for 3 min (or 0.5 mg ml^−1^ of TRDx when indicated), then excess bacteria were washed away with RB (30 times with 1 ml). Where indicated, 500 nM bafilomycin A_1_ (from a 100 μM stock in DMSO) was added to the macrophages for 1 h prior to infection and throughout the experiment.

Experiments used an inverted fluorescence microscope (Nikon TE300, Japan) equipped with transmitted light and a mercury arc lamp with epifluorescence illumination. To measure YFP and CFP fluorescence, two filter wheels (Lambda 10–2, Sutter Instruments, Novato, CA) held excitation filters (S500/20× and S436/10×, for YFP and CFP respectively, Chroma Technology Corporation, Rockingham, VT) and emission filters (S535/30m and S470/30m) with the dichroic mirror set (86002v1bs, Chroma Technology Corporation). A cooled CCD camera (Quantix Photometrics, Tucson, AZ) collected images and Metamorph software version 6.3 (Universal Imaging, West Chester, PA) controlled the equipment and image processing.

A ratio image was obtained by dividing each YFP image by the corresponding CFP image and multiplying by 1000. A binary mask was produced from the addition of the YFP and CFP images followed by application of a manual threshold. The binary and divided images were combined in a logical AND to produce ratio images that excluded non-cellular signals. A region was drawn around the phagosome in the ratio image and the average ratio of YFP/CFP in the phagosome was calculated. A second region drawn around the entire cell was used to measure the average fluorescence intensities of YFP and CFP over the entire cell. Relative ratios of YFP/CFP in the phagosome (R_p_) were then divided by the YFP/CFP for the entire cell (R_c_) to obtain a cell-normalized phagosome ratio (R_p_/R_c_).

### Measurement of vacuole perforation by *Lm*

In a method adapted from previous work (Shaughnessy *et al*., 2006), macrophages expressing PKCε-YFP were infected with a 100 μl mixture of wild-type *Lm*, TRDx (0.5 mg ml^−1^) and LY (0.5 mg ml^−1^) for 3 min. After infection, cells were washed thoroughly with RB. *Lm*-infected macrophages were located by phase-contrast optics. Using the 86006 dichroic filter set (Chroma Technology Corporation), four images were taken every minute for 30 min: phase-contrast, LY (exc. 436 nm/em. 535 nm), TRDx (exc. 580 nm/em. 630 nm) and YFP (exc. 492 nm/em. 535). The sequential loss of fluorescence from LY and TR was recorded relative to the timing of PKCε-YFP localization.

### Purification of LLO

Recombinant LLO was purified from *E. coli* strain BL21(DE3) transformed with the pET29b vector expressing LLO with a C-terminal six-histidine tag as previously described (Mandal and Lee, 2002). The protein yield was measured using the BCA assay (Pierce, Rockford, IL), and protein purity was analysed using SDS-PAGE. For some experiments, LLO was heat-inactivated at 70°C for 10 min. Haemolytic activity was measured using the sheep red blood cell-based haemolysis assay, as previously described (Mandal and Lee, 2002).

### Preparation of LLO liposomes

LLO/HPTS, hiLLO/HPTS, or HPTS liposomes were prepared with phosphatidylethanolamine (Avanti, Alabaster, AL) and cholesterylhemisuccinate (Sigma) in a 2:1 molar ratio using the thin film method (Lee *et al*., 1996; Mandal and Lee, 2002). LLO and HPTS were encapsulated inside liposomes at 0.25 mg ml^−1^ and 35 mM HPTS, respectively, in 30 mM Tris buffer, 100 mM NaCl, at pH 8.5, under non-reducing conditions. Liposomes underwent repeated sonication and freeze-thaw cycles. Unencapsulated protein and HPTS were removed by purification on a Sepharose CL-4B column (Amersham Pharmacia, Uppsala, Sweden).

### Osmotic lysis of pinosomes

Macrophage pinosomes were osmotically lysed according to previously published methods and adapted for this work (Okada and Rechsteiner, 1982; Moore *et al*., 1988). RAW macrophages expressing PKCε-YFP were exposed to hypertonic medium (0.5 M sucrose, 10% polyethylene glycol 1000, in RB) for 10 min (when stated, polyethylene glycol 1000 was not added). Coverslips were then washed five times and medium was replaced with hypotonic medium (60% RB and 40% water) for 3 min, then washed again and replaced with isotonic medium (RB). Phase-contrast and YFP images were taken after osmotic lysis.
